# Navigation-Assisted Micro-Window Excision of Thoracic Ossification of Ligamentum Flavum (Mishima Surgery) in Professional Baseball Pitchers: A Case Report and Technical Note

**DOI:** 10.3390/medicina59071303

**Published:** 2023-07-14

**Authors:** Ken Ishii, Norihiro Isogai, Ryunosuke Urata, Haruki Funao, Tatsuya Igawa, Hisanori Mihara, Tetsuya Yamazaki

**Affiliations:** 1 Department of Orthopaedic Surgery, Keio University School of Medicine, Tokyo 160-8582, Japan; 2 Department of Orthopaedic Surgery, Edogawa Hospital, Tokyo 133-0052, Japan; 3 New Spine Clinic Tokyo (Tentative), Tokyo 102-0093, Japan; 4 Society for Minimally Invasive Spinal Treatment (MIST), Tokyo 101-0063, Japan; 5 Spine and Spinal Cord Center, International University of Health and Welfare Mita Hospital, Tokyo 108-8329, Japan; ryu.urata62@gmail.com (R.U.); igatatsu.7@gmail.com (T.I.); 6 Department of Physical Therapy, School of Health Science, International University of Health and Welfare, Otawara 329-2763, Japan; 7 Department of Orthopaedic Surgery, Yokohama Minami Kyousai Hospital, Yokohama 236-0037, Japan; hmihara3838@gmail.com (H.M.); yamazaki@trio.plala.or.jp (T.Y.)

**Keywords:** ossification of the ligamentum flavum (OLF), thoracic spine, computed tomography (CT)-based navigation, navigation-assisted micro-window excision

## Abstract

*Background and Objectives*: Thoracic ossification of the ligamentum flavum (OLF) often causes myelopathy and/or radiculopathy. The disease is frequently observed in East Asian populations. Although thoracic OLF in young athletes who have underwent decompression surgery has been reported, the removal of posterior spinal bony elements and ligamentous complex may often cause postoperative thoracolumbar instability. We established a novel surgical technique that preserves the posterior spinal elements, including the spinous processes, facet joints, and supraspinous and interspinous ligaments for thoracic OLF. This is the first case report to describe a navigation-assisted micro-window excision of thoracic OLF. *Case:* A 32-year-old male right-handed professional baseball pitcher with significant weakness and numbness in the left leg was referred to our hospital. The patient was diagnosed with thoracic OLF at T10-11 based on radiographic and magnetic resonance images in August 2022. After exposure of the left T10-11 laminae via a small unilateral incision, the location of T10-11 OLF was detected over the lamina by O-arm navigation. Then, the micro-window was made directly above the OLF using a navigated air drill, and the OLF was removed on the ipsilateral side. The contralateral side of OLF was also resected through the same micro-window, achieving complete spinal cord decompression. *Results*: The next day of the surgery, his leg weakness and numbness were significantly improved. Six weeks after the surgery, he started pitching. Three months after surgery, his symptoms had gone completely, and he pitched from the mound. Approximately 6 months after surgery, he successfully pitched in a professional baseball game. *Conclusions:* A navigation-assisted micro-window excision of thoracic OLF effectively preserved the spinal posterior bony elements and ligamentous complex. However, long-term clinical outcomes should be evaluated in future studies.

## 1. Introduction

The ossification of the ligamentum flavum (OLF) is a relatively rare disease that often causes thoracic myelopathy and/or radiculopathy. Previous reports have indicated that either posterior decompression or posterior decompression and fusion has been widely performed [1,2,3,4]. Recently, a minimally invasive endoscopic technique has also been applied [5,6,7,8]. However, even minimally invasive endoscopic surgeries are prone to unintentionally damaging or breaking the posterior elements such as the facet joints, spinous processes, supraspinous, or interspinous ligaments [7,8,9,10]. Previous reports have described cases of myelopathy or radiculopathy due to thoracic OLF in baseball pitchers [11,12,13]. In these reports, the posterior decompression for the OLF was achieved at the thoracolumbar junction in all pitchers. However, decompression surgery, including laminectomy, laminotomy, and spinous processes-splitting laminectomy with the removal of OLF, can often cause postoperative instability at the thoracolumbar junction [14,15,16,17]. In this case report, we establish a novel surgical procedure that can enable us to resect thoracic OLF while preserving the posterior elements. The name for this surgical technique is as follows: “navigation-assisted micro-window excision”.

## 2. Case Report

### 2.1. Patient Presentation

**Figure 1:**
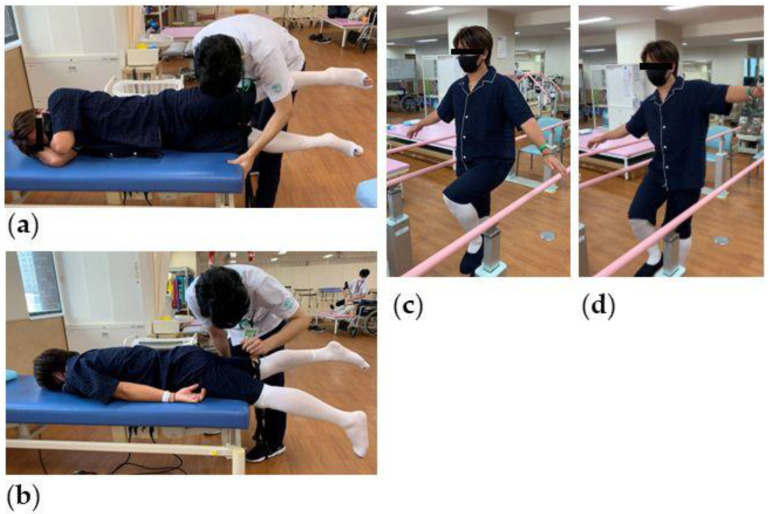
Preoperative physical examination. (**a**) Measurement of hip abduction strength. (**b**) Measurement of hip extension strength. In a single-leg standing test, in which the patient had to balance on one leg with his eyes closed, there was good stability on the right leg (**c**) but not on the left leg (**d**).

A 32-year-old male right-handed professional baseball pitcher began to feel weakness and numbness in his left leg in January 2022. His left leg “gave way” while pitching in May 2022. In August, he developed walking difficulties with progressive numbness and weakness in the left leg and was referred to our hospital. His neurological examination in the left lower extremity revealed an increased tone, 4/5 weakness throughout, and decreased sensation to light touch. His proprioception was slightly abnormal. The Chaddock and Babinski signs on the left side were positive, and no ankle clonus was observed. Although the patient was ambulatory, it was difficult for him to stand on his left leg with his eyes closed for a prolonged period of time. Preoperative physical examinations (e.g., range of motion [ROM], muscle strength, single-leg standing [Video S1], and circumference of leg) were performed (Figure 1). The pateint’s preoperative Japanese Orthopedic Association (JOA) score for thoracic myelopathy was 6 out of 11 points.

**Figure 2:**
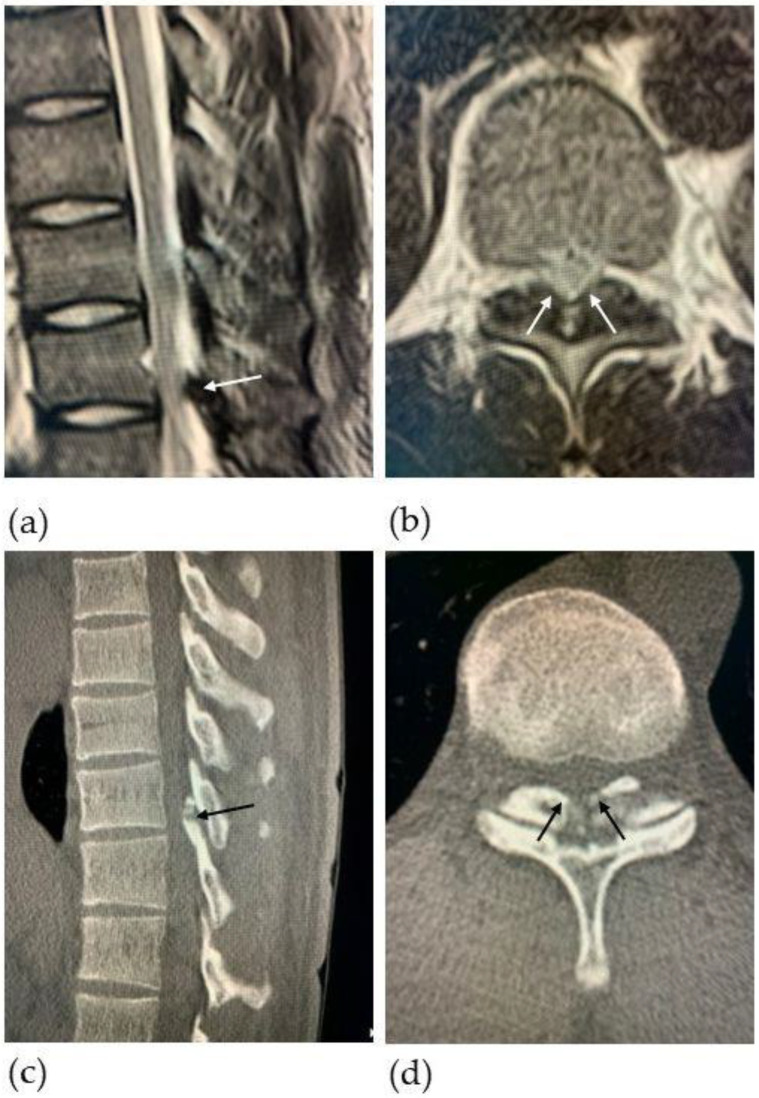
Preoperative magnetic resonance images (MRI) and computed tomography (CT) images. Sagittal (**a**) and axial (**b**) T2-weighted MRI of the thoracic spine show spinal cord compression with a high-intensity area at the T10-11 level due to a low-intensity signal lesion (white arrows). Sagittal (**c**) and axial (**d**) CT images of the thoracic spine show ossification of the ligamentum flavum (OLF) (black arrows), causing significant canal stenosis.

T2-weighted thoracic magnetic resonance imaging (MRI) revealed severe spinal cord compression with a low-intensity-signal mass (arrows in Figure 2a,b) and hyperintensity in the spinal cord at T10-11 level (Figure 2b). Subsequent computed tomography (CT) imaging was performed, confirming canal stenosis at T10-11, consistent with OLF (Figure 2c,d). Regarding the choice of surgical procedure, we believed that conventional surgery such as laminotomy and laminectomy may potentially cause postoperative instability at the thoracolumbar junction, as described previously in the literature [14,17,18]; thus, we sought out to identify a new surgical technique that could allow the thoracolumbar junction to withstand the force of torsion during pitching after decompression. We eventually proposed a new surgical technique consisting of navigation-assisted posterior OLF resection with the complete preservation of posterior elements, including the spinous processes, facet joints, and supraspinous and interspinous ligaments, which we subsequently named “navigation-assisted micro-window excision”.

### 2.2. Surgical Technique

**Figure 3:**
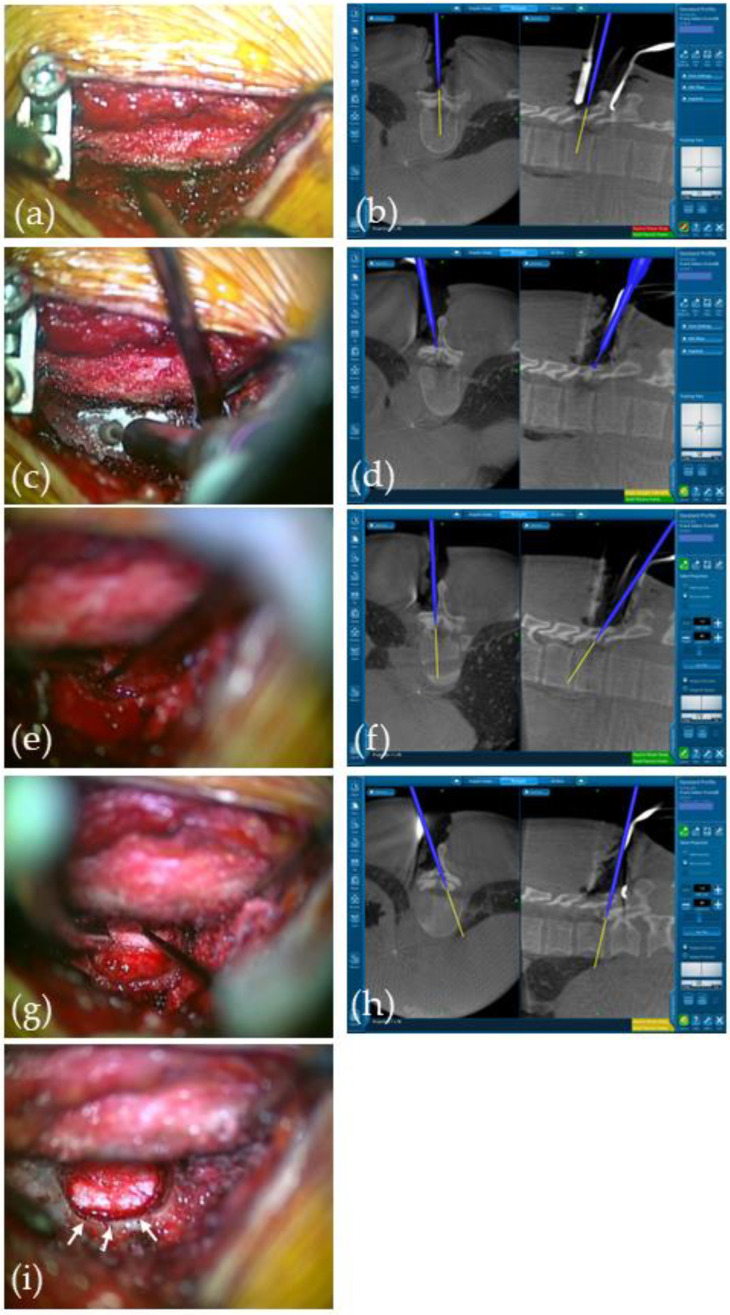
Intraoperative microscope views and O-arm navigation monitor images. Detecting the location of OLF over the T10 lamina using a navigation probe (**a**,**b**); the yellow lines in (**b**) indicate the target direction; drilling the surface of T10 lamina to make the micro-window using the navigation-assisted air drill (**c**,**d**); checking the edges of the dura matter on the ipsilateral side (**e**,**f**) and the contralateral side (**g**,**h**); decompressed dura mater and the spinal cord through the micro-window (white arrows in (**i**)).

The patient was positioned prone on the Jackson table. A skin incision of approximately 4 cm was made at the T10–11 level. Only the left paraspinal muscle was detached to expose the T9–10 spinous processes and T10–11 laminae. The reference arm was attached to the T9 spinous process, and the O-arm (Medtronic, Minneapolis, MN, USA) setting was changed to perform a CT scan within 15 sec in order to minimize the radiation dose. StealthStation S7 (Medtronic) was used as the navigation system. After confirming the location of the OLF from outside the spinal canal, the micro-window was created on the left side of T10 lamina just above the OLF using a Stealth-Midas high-speed drill (Medtronic) under a Leica M530 neurosurgical microscope (Leica, Wetzlar, Germany) (Figure 3 and Video S2). The drilling was carefully performed with a 4 mm diamond tip Midas Navidrill (Medtronic) to create a 13 mm craniocaudal × 12 mm wide micro-window. The T10 spinous process and T10–11 facets were completely preserved. The base of the T10 spinous process was also preserved to prevent postoperative fractures. The initial drilling was burred to the width of the dura mater within the spinal canal and to the craniocaudal length of the OLF as confirmed by the navigation system, achieving a flared “trumpet-shaped” micro-window. The use of the navigation system allowed for the precise positioning of the drill tip relative to the T10 lamina and OLF and confirmed the distance required to reach the spinal canal. Considering the potential for ossification and normal dura mater defects, the lesion was carefully shaved until an appropriate thickness was achieved and the ossification could be removed.

To drill the OLF of the contralateral side, the surgical bed was rotated to tilt the patient’s body to the right. After partially exposing the normal dura mater, the ossification was resected piece by piece with Kerrison rongeurs while carefully separating the shaved ossification and the normal dura mater. The left and right outer edges of the dura mater and the remaining shaved ossification of the craniocaudal area were resected using curved Kerrison rongeurs and small curettes. Epidural hemorrhage gradually increased as decompression was performed, and the pulsation of the dura mater was confirmed. The adequacy of the OLF extirpation was frequently checked using the navigation probe. Finally, a nerve hook was used to confirm that there was no pressure on the dura mater in the spinal canal (Video S2).

The surgery lasted for 185 min, and the estimated blood loss was 10 mL. We achieved OLF resection and complete decompression of the spinal cord through the micro-window and completely preserved the posterior spinal elements, including the spinous processes, facet joints, and supraspinous and interspinous ligaments.

### 2.3. Clinical Outcomes and Follow-Up

**Figure 4:**
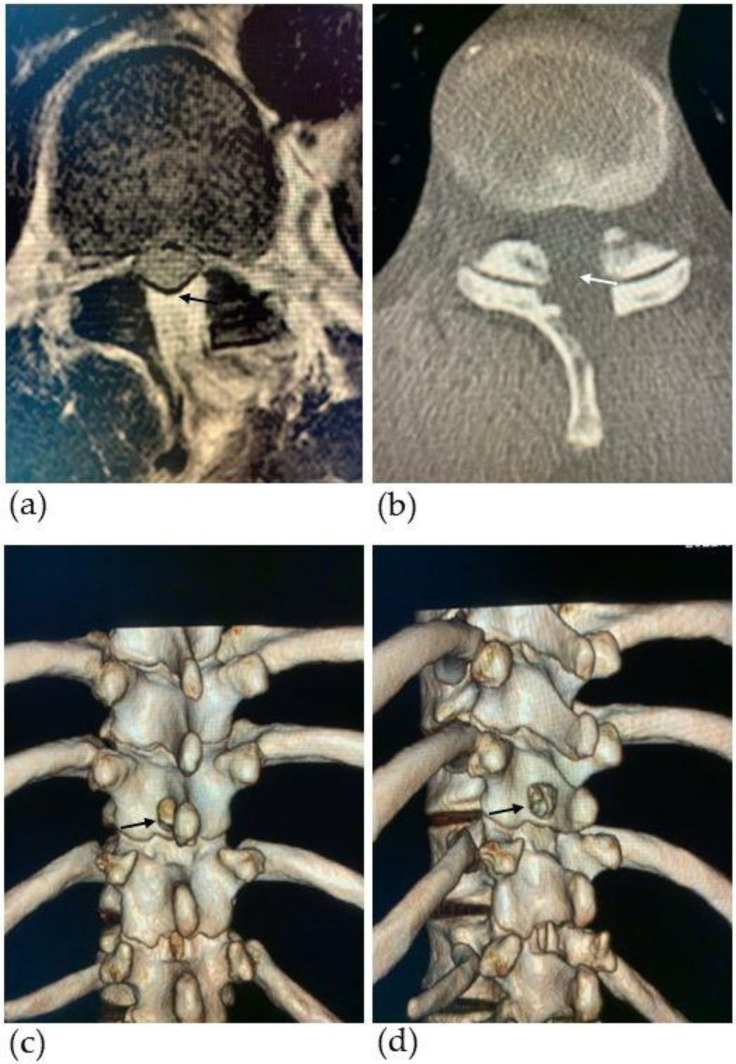
Postoperative magnetic resonance images (MRI) and computed tomography (CT) images. (**a**) Axial T2-weighted MRI of the thoracic spine at the T10-11 level shows spinal cord decompression (black arrow) with resection of ossification of the ligamentum flavum (OLF); (**b**) axial CT images of the thoracic spine at the T10-11 level shows resection of OLF (white arrow); the dorsal (**c**) and posterolateral (**d**) three-dimensional (3D) reconstruction CT images show a micro-window (black arrows) approximately 12 mm in diameter at the T10 lamina.

The patient was allowed to stand and walk the next day after the procedure. After reviewing the postoperative MRI and CT images, spinal cord decompression and the complete removal of OLF were confirmed (Figure 4). In the physical examination at 7 days after surgery, the patient showed a remarkable recovery in terms of hip ROM, hip strength, and in the one-leg standing stand (Table 1). Six weeks after the surgery, he started pitching (Video S3). Three months after surgery, his symptoms had almost completely gone, and he started to pitch from the mound (Video S4). At 6 months, he successfully pitched in a professional baseball game. Eight months after surgery, his JOA score significantly improved as he achieved a full score (11/11 points).

## 3. Discussion

Previously, various surgical techniques including posterior decompression (laminotomy, laminectomy) with/without fusion have been reported for thoracic OLF [1,2,3,4]. Especially in the case of high-level athletes, thoracolumbar decompression and fusion surgery may potentially end the patient’s career as an athlete due to the resulting limitations in spinal rotation. Therefore, decompression surgery is generally recommended instead of fusion surgery in athletes. However, the spinous processes, as well as the supraspinous and interspinous ligaments, are often sacrificed in such surgeries.

In recent years, unilateral posterior decompression using micro-endoscopic or full-endoscopic surgeries have also been reported in the literature. Even in unilateral endoscopic surgeries, either spinous processes, supraspinous, or interspinous ligaments are often sacrificed during surgery, or they involve unintentional fractures [6,7,8,9]. In addition, minimally invasive surgery is preferred by some physicians for the treatment of thoracic myelopathy, but the surgery is technically demanding [18,19]. Degreif et al. [20] reported a comparative experimental study using human vertebral columns of rotational stability at the thoracolumbar spine after interlaminar ultrasound window, hemilaminectomy, and laminectomy. They indicated that a laminotomy of up to 10 × 20 mm in size can cause a 6% loss in stability. A significant decrease in rotational stability is caused by a hemilaminectomy (20% loss), and a laminectomy cause a 27% loss in stability compared to those with an intact spine. Therefore, it is important to preserve the posterior bony and ligamentous elements during thoracic OLF resection, especially in high-level athletes. Moreover, the rotational instability of the spine causes severe pain and often must be treated by spondylodesis [20]. In addition, many previous reports have shown that instability at the thoracolumbar junction induces osteoarthritis in the facet joints [14,15,16]. Removing the spinous processes and lamina may not cause immediate instability, but progressive angular deformity does occur in certain individuals.

The advantages of our novel surgical technique not only include the preservation of the spinal posterior elements but also extend to the use of CT-based navigation, which allows for accurate identification of the OLF from a high position directly above the lamina, leading to the ipsilateral creation of a micro-window of the lamina and enhancing one’s ability to resect the OLF alone by burring a “trumpet-shaped” window. CT-based navigation has been introduced to ensure accurate and effective implant placement [21] and provides surgeons with more confidence by providing a three-dimensional (3D) visualization of the skeletal anatomy that is not clear through surgical exposure alone, especially in complex spine surgery [5,22]. To the best of our knowledge, this is the first case report to describe a navigation-assisted posterior OLF resection while completely preserving posterior elements, including the spinous processes, facet joints, and supraspinous and interspinous ligaments. We have proposed a novel surgical technique for thoracic OLF, which is named “navigation-assisted micro-window excision (Mishima Surgery)”. This technique allows the patient to maintain a normal spinal structure and rotational stability in the thoracic and lumbar regions of the spine. Shortly after surgery, the muscle weakness, painful sensations, and proprioception in the left leg almost all completely improved. This season, he was registered to play in the opening game of the Nippon Professional Baseball (NPB) league season, helped his team to record three wins in April 2023, and was selected as the most valuable player of the month. No symptoms due to spinal rotational instability were observed at 8 months after surgery. On the other hand, the main purpose of this surgical procedure was for the patient to return to competition. Because the procedure decompresses the dural tube via the micro-window, there is a possibility of recurrent myelopathy due to recurrence of OLF in the future, and careful follow-up support is needed.

Thoracic OLF is typically seen in males in their 60s to 70s [23,24], while young adults are rarely affected [25]. Mechanical stress has been proposed to accelerate hypertrophy in the ossification of the yellow ligament (OYL) [11,26]. Yoshida et al. [27] suggested that the yellow ligament is hypertrophied by mechanical stress and that the main constituent of hypertrophy is the proliferation of type 2 collagen at the enthesis. It has also been suggested that mechanical stress induces hypertrophy of the yellow ligament and aggravates this condition, leading to ossification. Previous case reports of thoracic OLF in young baseball pitchers have been reported. These reports inferred that the asymmetrical rotatory mechanical stress would affect the development of OLF [11,12]. In our case, no previous generalized disorders or any thoracic injuries, including ring apophyseal fractures, were found. Furthermore, there was no previous history of treatment for OLF or the ossification of posterior longitudinal ligaments in his family. The patient in this study is a professional baseball pitcher, and we speculate that he had endured excessive mechanical stress due to repeated torsion and anteroposterior flexion of the spine for a prolonged period since childhood. We believe that the thoracolumbar junction, a bony cage-like structure located directly below the thorax, might have experienced acute and chronic inflammation due to a large concentration of mechanical stress, resulting in the occurrence of OLF. A total of six high-level baseball pitchers with thoracic OLF have been previously reported [11,12,13,28] in addition to our case (Table 2). All five cases of myelopathy were right-handed pitchers. Surprisingly, the main lesion of OLF was located on the left side in all cases, and myelopathy of the left lower extremity was observed. Laudner et al. [14] analyzed thoracolumbar range of motion in baseball pitchers and infielder/outfielder players. The study included 56 asymptomatic collegiate and minor-league baseball pitchers and 42 infielder/outfielder players. Pitchers had a statistically greater amount of rotation ROM towards the non-throwing arm compared to infielder/outfielder players (51.9 vs. 47.7 degrees). Pitchers also had a statistically greater amount of rotation ROM in their non-throwing arm compared to their throwing side (51.9 vs. 48.8 degrees). In a volunteer study of thoracolumbar rotation, right-handed pitchers had a greater left rotation angle than right rotation angle, and left-handed pitchers had a greater right rotation angle than left rotation angle. Therefore, if repetitive rotational stress is a factor in the development of OLF, we can infer that right-handed pitchers are more likely to develop OLF on the left side of the thoracolumbar junction while left-handed pitchers are more likely to develop OLF on the right side.

## 4. Conclusions

Thoracic OLF is often observed in high-level baseball pitchers in East Asian countries. Surgical procedures that reduce postoperative spinal instability should be chosen. In the current study, we have proposed a novel surgical technique for thoracic OLF, which is named “navigation-assisted micro-window excision”. This procedure successfully preserved the spinal posterior bony elements and ligamentous complex. However, long-term clinical outcomes should be evaluated in future studies.

## Figures and Tables

**Table 1:** Results from the pre- and post-operative physical examinations.

Type of Examination			Preoperative		At Discharge		1 Months Post-op		3 Months Post-op	
			Right	Left	Right	Left	Right	Left	Right	Left
ROM (degrees)	Hip	flexion	120	120	120	120			120	120
		extension	20	20	20	20			20	20
		abduction	35	35	45	45			45	50
		internal rotation	45	45	45	45			30	35
		external rotation	40	35	35	40			40	35
		straight leg raising	70	60	70	70	70	80	60	70
Maximum muscle strength (kg)		back muscle	114.5				124.5		148	
		grip power	43.4	32.2	47	40.4	45.2	41.9	43.1	39.9
Muscle strength (kgf)	Hip	extension	46.2	29.4	37	36.3	61.3	51.8	57.7	55.4
		abduction	19.1	12.7	34	33.5	48.1	49.7	44.7	48
	Knee	extension	54.3	33.2	65.4	69.7	69.7	68	67	69.7
Circumference (cm)	Thigh		46	46	53.5	55			57	57.5
	Lower thigh		42	42	39.5	39			39.5	39
Single-leg standing with eyes closed (s)			50.84	25.85	60	60			60	60

Abbreviations: ROM, range of motion; kg, kilogram; kgf, kilogram-force; s, seconds. op, operation; Muscle strength in the back muscles and grip power were measured twice; the higher strength result was accepted. The single-leg standing with eyes closed test was also conducted twice, and the longer time was accepted.

**Table 2:** Summary of baseball pitchers with thoracic ossification of the ligamentum flavum.

Reference	Year	Age (y/o), Sex	Pitch Style	Symptoms	Duration of Symptoms (mo)	Levels of OLF	Laterality of OLF	Surgical Procedures	F-U (mo)	Time of Pitching
Kaneyama [11]	2008	28, M	Rt	Left leg weakness, numbness on the bottom of both feet	2	T10–12	Lt > Rt	Laminectomy at T10–12	12	
Kaneyama [11]	2008	24, M	Rt	Left leg weakness		T10–12	Lt > Rt	Laminectomy at T10, laminoplasty at T11	Over 6	6 months
Tadokoro [28]	2017	28, M	Rt	Left > right legs numbness and urinary dysfunction	2	T6–7, T11–12	Bilateral, Lt > Rt	Laminectomy and fusion at T6–7 and T11–12	18	12 months
Kato [12]	2021	27, M	Lt	Left chest and upper abdominal pain and numbness		T8–12	Lt > Rt	Physiotherapy	108	4 months
Kato [12]	2021	22, M	Lt	Pain in the left lower ribs	24	T8–12	Lt > Rt	Physiotherapy	Over 2	6 weeks
Kato [13]	2021	28, M	Rt	Left buttock and thigh pain	12	T10–12	Lt > Rt	Laminectomy at T10–12	16	4 months
Our case	2023	32, M	Rt	Left leg weakness and numbness	7	T9–11	Lt > Rt	Navigation-assisted micro-window excision	9	6 weeks

Abbreviations: y/o, years old; M, male; Rt, right; Lt, left; mo, months; OLF, ossification of the ligamentum flavum; T, thoracic; F-U, follow-up.

## Data Availability

All relevant data are included in this paper.

## Supplementary Materials

The following supporting information can be downloaded at: https://www.mdpi.com/article/10.3390/medicina59071303/s1, Video S1: Preoperative single-leg standing test with eyes closed; Video S2: Intraoperative images (macroscope, microscope, and navigation images); Video S3: Six-week postoperative pitching; Video S4: Three-month postoperative pitching.
